# Acute cecal volvulus: A diagnostic and therapeutic challenge in emergency: A case report

**DOI:** 10.1016/j.amsu.2019.10.021

**Published:** 2019-10-31

**Authors:** Abdoul Aliou Zabeirou, Houssam Belghali, Tarek Souiki, Karim Ibn Majdoub, Imane Toughrai, Khalid Mazaz

**Affiliations:** a Department of General and Visceral Surgery, Hassan II University Hospital of Fez, 30050, Fez, Morocco; b Faculty of Medicine and Pharmacy, Sidi Mohamed Ben Abdellah University of Fez, 30050, Fez, Morocco

**Keywords:** Cecal volvulus, Bowel obstruction, Manual untwisting, Caecopexy

## Abstract

**Introduction:**

Cecal volvulus is an uncommon cause of intestinal obstruction due to an axial twist of the caecum, ascending colon and terminal ileum around the mesenteric pedicle. It is responsible for 1%-1.5 of all intestinal obstructions in adult. The clinical signs may be highly variables and can be responsible of delays in diagnostic and treatment. The delay in diagnosis leads to intestinal necrosis or perforation. The mortality ranges from 10 to 40% depending on the presence of a viable or gangrenous intestine.

**Presentation of case:**

A 64 year old woman admitted the emergency department for acute bowel obstruction. Clinical examination found typically acute bowel obstruction signs. Plain radiography showed dilated gas-filled segment of the colon in the left side of abdomen and volvulus of cecum was suspected. Enhanced abdominal CT scan confirmed the diagnosis. Emergency exploratory laparotomy was performed and confirmed the cecal volvulus. A manual untwisting of volvulus and a Caecopexy were performed. The patient subsequently recovered uneventfully and was discharged on postoperative day 3.

**Discussion:**

The management of cecal volvulus requires prompt (emergency) diagnosis and prompt surgical intervention. Any delay in diagnosis may lead to intestinal necrosis or perforation and worsening the prognosis in patients who are generally elderly. Several authors reported a high mortality rate of cecal volvulus due to delay to diagnosis and surgical intervention.

**Conclusion:**

The low incidence of this condition needs a high index of suspicion and emergency surgical management. Despite significant progress in medical imaging, the preoperative diagnosis of cecal volvulus is very difficult. As a result, the treatment is often delayed.

## Highlights

• Cecal volvulus is an uncommon cause of intestinal obstruction with a high rate of mortality.
• Clinically it is difficult to differentiate cecal volvulus from other forms of bowel obstruction.
• The delay in diagnosis is responsible of high rate of mortality in this clinical condition.
• The “coffee bean”, “bird beak” and “whirl” signs are pathoneumonic for cecal volvulus when visualized on CT scan.
• Caecum Volvulus requires immediate surgery.

## 1 Introduction

Cecal volvulus is an axial twisting of the cecum, involving terminal ileum and ascending colon du to absence of normal cecal fixation [1]. The First description of cecal volvulus was made by Rokitansky in 1837 [2]. It is responsible for 25%–40% of all volvulus of colon in adults and represents the second part of the colon that is most commonly affected by the volvulus after sigmoid colon [3]. Clinical signs of cecal volvulus are not specific, and it difficult to differentiate cecal volvulus from other forms of bowel obstruction [4]. Diagnosis may be delayed and acute cecal volvulus may progress to cecal gangrenous and its perforation leading to the acute peritonitis. Abdominal X-ray and abdominal CT scan are the essential radiological procedures in the diagnosis of volvulus of the cecum [5]. The only effective treatment for cecal volvulus is surgical intervention [6].

We report a case of A 64 year old woman patient admitted to the emergency department for bowel obstruction. The diagnosis of cecal volvulus was confirmed by CT scan. Laparotomy and Caecopexy were performed. The postoperative course was uneventful. This case is an exceptional case of a patient with history of mobile cecum syndrome, admitted for cecal volvulus diagnosed preoperatively.

The case report has been reported in line with the SCARE criteria [7].

## 2 Case presentation

A 64 year old woman patient, with history of chronic colicky abdominal pain and intermittent bowel subocclusion; admitted in the emergency Department for acute and diffuse abdominal pain of about 5 hour duration. She also complained of nausea and bilious vomiting. Clinical examination found a patient hemodynamically stable, apyretic and dehydrated. Abdominal examination revealed diffuse abdomen distension. Abdominal palpation identified a firm and tympanitic mass in periumbilical region, and diffuse tenderness without peritoneal irritation signs was noted. Laboratory tests showed a functional renal failure, hyponatremia and hypokalemia. White Blood Cell and C-reactive protein were normal.

**Fig. 1:**
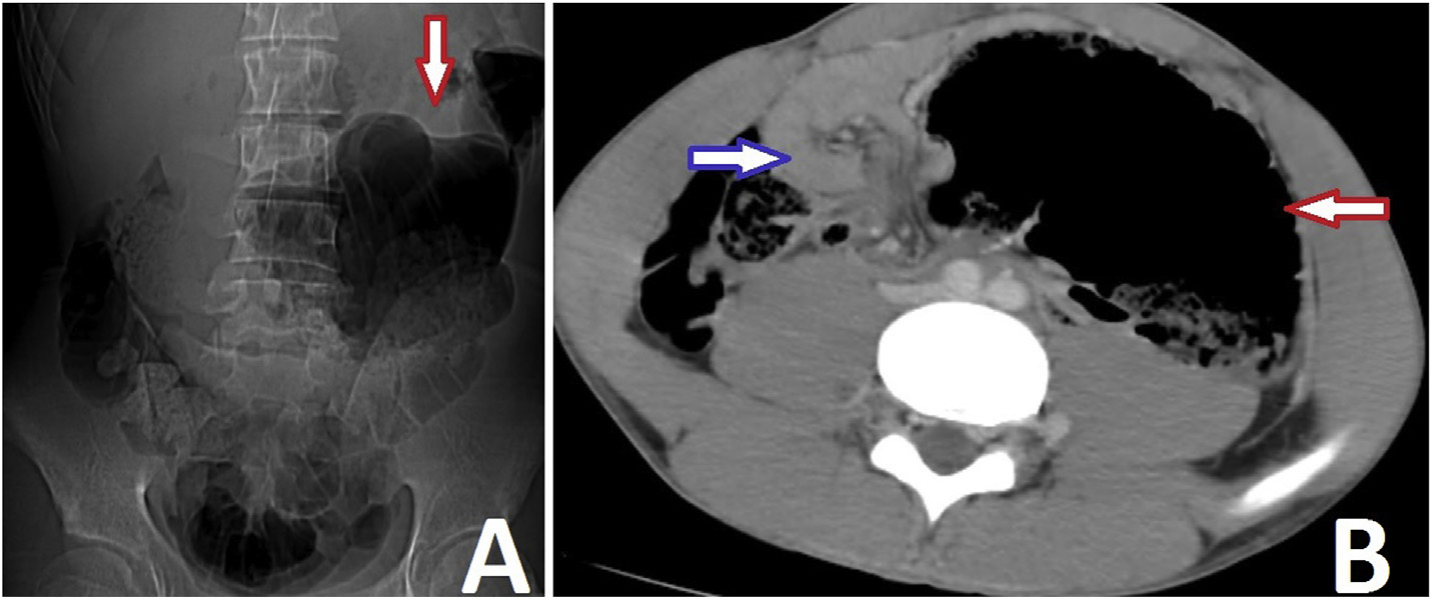
A: Plain radiography revealed a markedly air-distended bowel in the left abdomen, B: Abdominal CT scan showing coffee bean sign (red arrow) and whirl sign (blue arrow). (For interpretation of the references to colour in this figure legend, the reader is referred to the Web version of this article.)

Plain radiography showed dilated gas-filled segment of the colon in the left side of abdomen (Fig. 1). An abdominal CT showed an axial twisting of the ascending colon and terminal ileum resulting in closed loop obstruction of the caecum. The grossly dilated air-distended caecum was inverted and occupied the left upper quadrant of the abdomen. CT showed also the mesenteric whirl signs (Fig. 1).

**Fig. 2:**
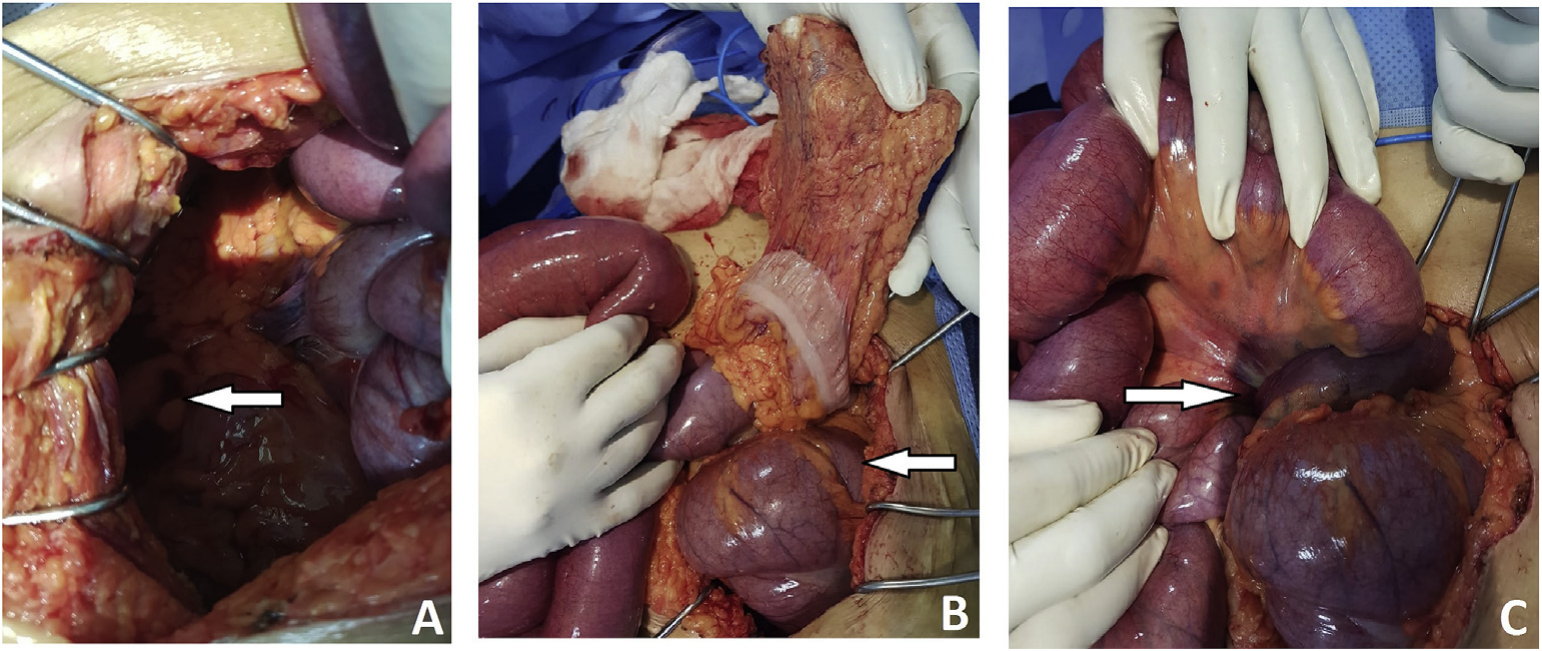
Intraoperative view showing; A: empty right iliac fossa; B: distended caecum occupied the left upper quadrant of the abdomen (arrow); C: cecal volvulus with the point of twisting for the caecum and terminal ileum being visible (arrow).

**Fig. 3:**
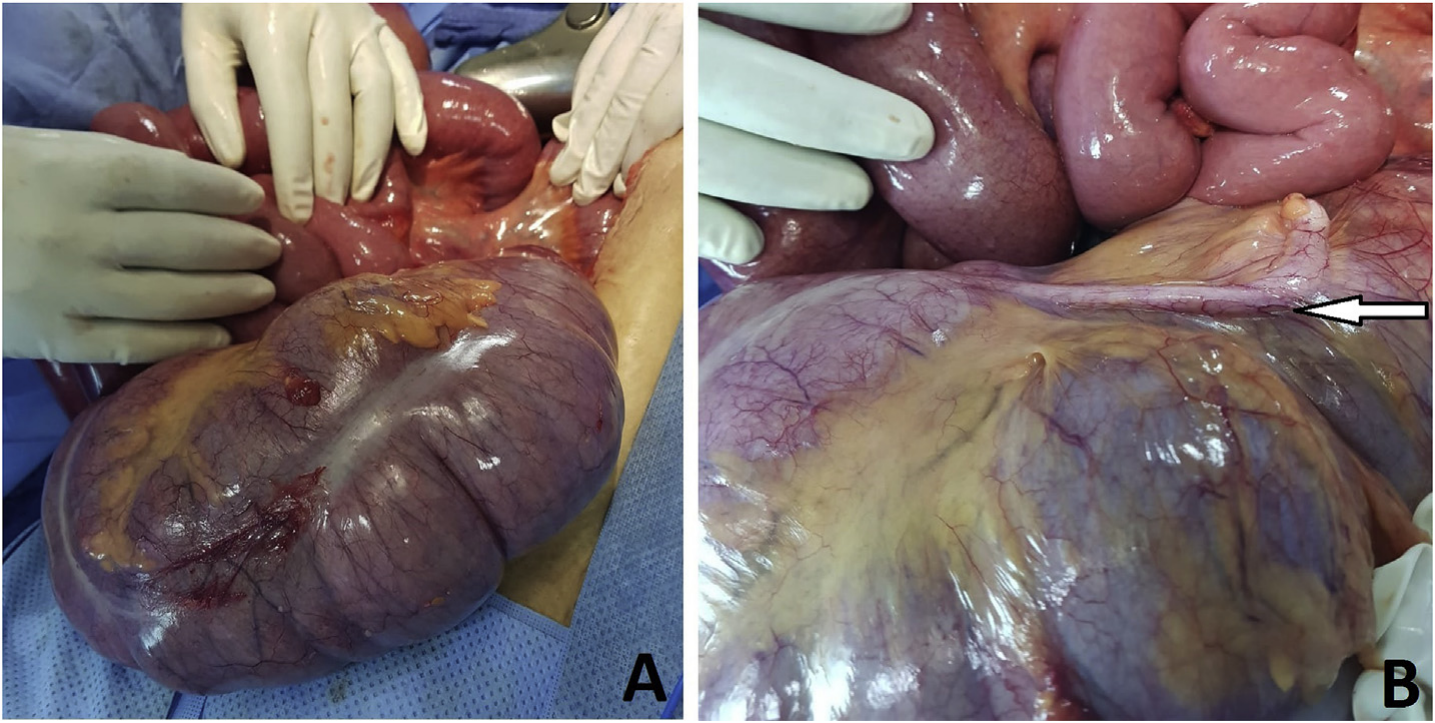
Intraoperative view showing; A: unfixed Cecum to the retroperitoneum after manual untwisting. B: Appendix (arrow).

An emergency laparotomy was performed. Surgical exploration revealed that the right iliac fossa was empty; and cecal volvulus involving ascending colon and terminal ileum (Fig. 2). There was no intestinal necrosis or perforation. A manual untwisting counterclockwise of volvulus in 360° (Fig. 3) and A Caecopexy was performed.

She subsequently recovered uneventfully and was discharged on postoperative day 3. She remains free of recurrence 23 months after surgery.

## 3 Discussion

Cecal volvulus is an infrequently encountered clinical condition and an uncommon cause of intestinal obstruction [8]. It is responsible for approximately 1–1.5% of all intestinal obstructions, while 20–40% of all colonic volvulus. Its incidence is 2.8–7.1 cases per million annually [3]. Cugnenc et al. reported in a series an average age of occurrence of cecal volvulus at 61.8 years and there is no predisposition related to sex established [9].

Cecal volvulus occurs in case of inadequate right colon fixation or congenital abnormalities in which the right colon does not properly fuse with the lateral peritoneum [10]. Based on reports from necropsy reviews, sufficient cecal mobility for volvulus and bascule formation is found in 11% and 25% of adults, respectively [10]. In addition of cecal fixation abnormalities, the cecal volvulus can occurs secondary to adhesions from abdominal surgery, chronic constipation, pregnancy or prolonged immobility [11].

The Mobile caecum syndrome has been reported to occur in nearly 50% of patients before the onset of acute volvulus [12]. Typically, the patients have recurrent symptoms consisting of generalized or localized right lower quadrant abdominal pain, abdominal distension, and pain resolution after the passage of flatus. The physical findings in patients during symptomatic episodes may include high pitched bowel sounds and right lower quadrant abdominal tenderness. However, these abnormal physical findings generally disappear as the patients’ symptoms resolve [13]. In stage of acute volvulus, the patient presents typically an acute bowel obstruction signs and it is difficult to differentiate cecal volvulus from other forms of small bowel obstruction. A tender and dilated caecum may be palpable in skinny patient, and can help to differentiate cecal volvulus from other forms of bowel obstruction [14]. In case of Delay or missing in the diagnosis; untreated acute cecal volvulus may progress to intestinal gangrenous and perforation leading to the acute stercoral peritonitis [14]. With this clinical presentation, patients typically exhibit severe abdominal pain, peritoneal irritation, consciousness disorder, and hemodynamic instability [15].

Abdominal radiography may allow a diagnosis if three typical signs are present: caecum dilatation; a single air-fluid level in the right lower quadrant; and absence of gas in the colon. However, up to 30% of patients do not show these radiographic features [16]. Young et al. emphasized that the important distinction between cecal volvulus and sigmoid volvulus is that haustra in the intestinal wall are often seen in case of cecal volvulus, whereas haustra are not visible in case sigmoid colon volvulus [17]. Barium enema has been applied for cecal volvulus confirmation, with reported diagnostic accuracy of 88% for acute cecal volvulus. It has occasional success in reduction of volvulus [18]. The barium enema also enables to visualization of the distal colon for the exclusion of coexisting abnormality that may have contributed to the cecal volvulus formation. The barium enema is not recommended for the evaluation of patients with suspected perforation and gangrenous bowel [18]. CT scan replaced barium enema as the preferred imaging modality for the diagnosis of acute cecal volvulus [5]. The three pathoneumonic CT signs associated with acute cecal volvulus are: ‘‘coffee bean’’, ‘‘bird beak’’, and ‘‘whirl signs’’. In the setting of acute cecal volvulus, the whirl is composed of spiralled loop of collapsed caecum, with low attenuating fatty mesentery and engorged mesenteric vessels. Visualization of a gas filled appendix is also a CT scan sign associated with cecal volvulus. The CT scan may show signs of intestinal ischemia or necrosis, which manifest as submucosal edema, diminished or non-enhancement of intestinal wall, pneumatosis intestinalis or signs of intestinal perforation such as pneumoperitoneum [5].

Several authors have reported successful colonoscopic reduction of cecal volvulus [19]. The success rate for colonoscopic reduction of cecal volvulus is only 30% and given the potential for colonic perforation colonoscopy is not advised in the management of cecal volvulus.

It is generally agreed that the only effective treatment for cecal volvulus is surgical intervention. Surgical options include manual detorsion, caecopexy, caecostomy, and right colectomy by open or laparoscopic approaches [6]. If intestinal gangrenous and perforations are encountered, the non-viable intestines should be resected. In the presence of a viable bowel, detorsion and Caecopexy has been proposed as a relatively safe procedure, but it has also been associated with a high recurrence rate [20].

Laparoscopic procedures are being increasingly used to manage cecal volvulus. Several reports of laparoscopic treatment of cecal volvulus were published [6].

## 4 Conclusion

The volvulus of the cecum occurs on a mobile cecum following an axial twisting of the cecum around its mesentery. The diagnosis is most often delayed because of clinical signs that are nonspecific. Abdominal CT is the key examination for diagnosis. The treatment is only surgical. The diagnostic delay is responsible of the high mortality rate reported in literature.

## Ethical approval

There is no ethical committee in our country (Not applicable for this Manuscript).

## Sources of funding

No sources of funding.

## Author contribution

All authors contributed to the study design. AZ and HB were involved in clerking the patient, collecting Preliminary data, performed the surgery and wrote the manuscript draft.ST, KI, IT and KM readied and corrected the manuscript. All authors read and approved the final manuscript.

## Registration of research studies

Is a case report.

## Guarantor

Abdoul Aliou Zabeirou.

## Consent for publication

Written informed consent was obtained from the patient for publication of this case report and any accompanying images. A copy of the written consent is available for review by the Editor-in-Chief of this journal.

## Provenance and peer review

Not commissioned, externally peer reviewed.

## Declaration of competing interest

All authors declare that they have no any conflicts of interest.
